# Low-Concentration PM_2.5_ and Mortality: Estimating Acute and Chronic Effects in a Population-Based Study

**DOI:** 10.1289/ehp.1409111

**Published:** 2015-06-03

**Authors:** Liuhua Shi, Antonella Zanobetti, Itai Kloog, Brent A. Coull, Petros Koutrakis, Steven J. Melly, Joel D. Schwartz

**Affiliations:** 1 Department of Environmental Health, Harvard T.H. Chan School of Public Health, Boston, Massachusetts, USA; 2 Department of Geography and Environmental Development, Ben-Gurion University of the Negev, Beer Sheva, Israel; 3 Department of Biostatistics, Harvard T.H. Chan School of Public Health, Boston, Massachusetts, USA

## Abstract

**Background:**

Both short- and long-term exposures to fine particulate matter (≤ 2.5 μm; PM_2.5_) are associated with mortality. However, whether the associations exist at levels below the new U.S. Environmental Protection Agency (EPA) standards (12 μg/m^3^ of annual average PM_2.5_, 35 μg/m^3^ daily) is unclear. In addition, it is not clear whether results from previous time series studies (fit in larger cities) and cohort studies (fit in convenience samples) are generalizable.

**Objectives:**

We estimated the effects of low-concentration PM_2.5_ on mortality.

**Methods:**

High resolution (1 km × 1 km) daily PM_2.5_ predictions, derived from satellite aerosol optical depth retrievals, were used. Poisson regressions were applied to a Medicare population (≥ 65 years of age) in New England to simultaneously estimate the acute and chronic effects of exposure to PM_2.5_, with mutual adjustment for short- and long-term exposure, as well as for area-based confounders. Models were also restricted to annual concentrations < 10 μg/m^3^ or daily concentrations < 30 μg/m^3^.

**Results:**

PM_2.5_ was associated with increased mortality. In the study cohort, 2.14% (95% CI: 1.38, 2.89%) and 7.52% (95% CI: 1.95, 13.40%) increases were estimated for each 10-μg/m^3^ increase in short- (2 day) and long-term (1 year) exposure, respectively. The associations held for analyses restricted to low-concentration PM_2.5_ exposure, and the corresponding estimates were 2.14% (95% CI: 1.34, 2.95%) and 9.28% (95% CI: 0.76, 18.52%). Penalized spline models of long-term exposure indicated a larger effect for mortality in association with exposures ≥ 6 μg/m^3^ versus those < 6 μg/m^3^. In contrast, the association between short-term exposure and mortality appeared to be linear across the entire exposure distribution.

**Conclusions:**

Using a mutually adjusted model, we estimated significant acute and chronic effects of PM_2.5_ exposure below the current U.S. EPA standards. These findings suggest that improving air quality with even lower PM_2.5_ than currently allowed by U.S. EPA standards may benefit public health.

**Citation:**

Shi L, Zanobetti A, Kloog I, Coull BA, Koutrakis P, Melly SJ, Schwartz JD. 2016. Low-concentration PM_2.5_ and mortality: estimating acute and chronic effects in a population-based study. Environ Health Perspect 124:46–52; http://dx.doi.org/10.1289/ehp.1409111

## Introduction

Many studies have found associations between fine particulate matter [PM with aerodynamic diameter ≤ 2.5 μm (PM_2.5_)] and increased mortality (Dockery et al. 1993; Franklin et al. 2007; Pope et al. 2002; Schwartz 1994; Zanobetti and Schwartz 2009). Biological evidence has been established for plausible mechanisms between PM_2.5_ and mortality, such as increased risk of ventricular arrhythmia and thrombotic processes, increased system inflammation and oxidative stress, increased blood pressure, decreased plaque stability, and reduced lung function, among others (Brook et al. 2009; Gauderman et al. 2004; Gurgueira et al. 2002; Suwa et al. 2002; Yue et al. 2007). Based on evidence from epidemiological and toxicological studies (Chen and Nadziejko 2005; Furuyama et al. 2006; Ohtoshi et al. 1998), National Ambient Air Quality Standards (NAAQS) were implemented for fine particulate matter. For example, the U.S. Environmental Protection Agency (EPA) revised the fine particle NAAQS in 1997, 2006, and 2012 in order to protect public health (U.S. EPA 1997, 2006, 2013). Further changes in the standards require additional studies to elucidate whether health effects occur at levels below the current annual and daily U.S. EPA NAAQS of 12 and 35 μg/m^3^, respectively. The Clean Air Act Amendments of 1990 require the U.S. EPA to review national air quality standards every 5 years to determine whether they should be retained or revised; thus, whether health effects can be observed below the current standards is of great interest and importance.

Previous studies have generally focused on either long-term (Hart et al. 2011; Jerrett et al. 2005; Puett et al. 2009; Schwartz 2000) or short-term (Dominici et al. 2006; Katsouyanni et al. 1997; Samoli et al. 2008; Schwartz and Dockery 1992) exposures across the entire range of PM_2.5_ concentrations. In the case of time series analyses of short-term exposures, the need to ensure the relevance of the monitoring data as well as the need to have a study population of a size for sufficent power has limited analyses to large cities; hence, exurbs, small cities, and rural areas are not generally represented in the literature, which may compromise the generalizability of the results. In addition, there is spatial variability in PM_2.5_ concentrations within cities that time series studies generally do not take into account, which can introduce exposure measurement error (Laden et al. 2006; Lepeule et al. 2012).

Chronic effects studies began using comparisons across cities of mortality experiences of cohorts living in various communities and the monitored air pollutant concentrations in those communities (Dockery et al. 1993; Pope et al. 1995). Again, these studies suffered from exposure error due to failure to capture within-city spatial variability in exposure. Because the geographic exposure gradient is the exposure contrast in these studies, the failure to capture within-city contrasts leads to classical measurement error with expected downward bias. Studies with, for example, land use regression estimates of exposure have generally reported larger effect sizes (Miller et al. 2007; Puett et al. 2009). Previous cohort studies have not controlled for the acute effects of particles when estimating chronic effects, raising the question of whether there are independent chronic effects that represent more than the cumulative effects of acute responses.

In general, existing study cohorts are not representative of the overall population. For example, the American Cancer Society (ACS) cohort has a higher level of education than the U.S. population as a whole (Stellman and Garfinkel 1986). Hence, few population-based cohort studies have been conducted until recently (Kloog et al. 2013).

Several time series studies examined the concentration–response relationship between PM_2.5_ and mortality below concentrations of 100 μg/m^3^; these studies generally reported a linear concentration–response relationship (Samoli et al. 2008; Schwartz and Zanobetti 2000). However, there have been few studies focusing on exposures below the current daily U.S. EPA standard of 35 μg/m^3^.

Many studies have examined the shape of the concentration–response curve for long-term exposure versus short-term exposure, but in general, they have not covered population-based cohorts, or have only included very low exposures (Schwartz et al. 2008; Crouse et al. 2012).

We recently presented a new hybrid method of assessing temporally and spatially resolved PM_2.5_ exposure for epidemiological studies by combining 1 km × 1 km resolution satellite-retrieved aerosol optical depth (AOD) measurements with traditional land use terms, meteorological variables, and their interactions (Kloog et al. 2014a). This approach allows for predicting daily PM_2.5_ concentrations at a 1 km × 1 km spatial resolution throughout the New England area of the northeastern United States. We also validated our model’s performance in rural areas: 10-fold cross-validation (CV) of our model in rural areas (using the IMPROVE stations) resulted in a CV *R*^2^ of 0.92. Further details have been published (Kloog et al. 2014a).

The present study aimed to simultaneously estimate acute and chronic health effects of PM_2.5_ in a population-based Medicare cohort (≥ 65 years of age) encompassing the New England region. We used high-spatial-resolution exposure estimates based on satellite measurements that are available across the region and not just in limited locations. To make this study relevant to future assessments of current U.S. EPA standards, we repeated the analysis after restricting the data to long-term exposures (365-day moving average) < 10 μg/m^3^ and repeated the time series analysis of short-term exposures after restricting the data to 2-day average exposures < 30 μg/m^3^.

## Methods

**Figure 1:**
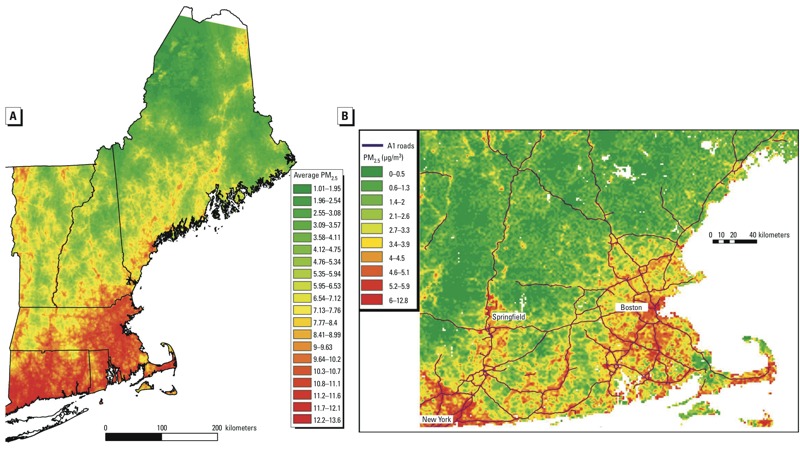
(*A*) Mean PM_2.5_ concentrations in 2004 at a high resolution (1 km × 1 km) across New England predicted by the AOD models. (*B*) Predicted PM_2.5_ concentrations at a 1 km × 1 km grid for 15 November 2003.

*Study domain.* The spatial domain of our study included the New England area, comprising the states of Connecticut, Maine, Massachusetts, New Hampshire, Rhode Island, and Vermont (Figure 1A).

*Exposure data.* A 3-stage statistical modeling approach for predicting daily PM_2.5_ was previously reported incorporating AOD and land use data for the New England region (Kloog et al. 2011). Previous studies have shown that using actual physical measurements in our prediction models improved predictive accuracy over that of comparable land use or spatial smoothing models (Kloog et al. 2011). With AOD retrieved by the multi-angle implementation of atmospheric correction (MAIAC) algorithm, a similar approach was applied for estimating daily PM_2.5_ exposures in New England at a spatial resolution of 1 km × 1 km (Kloog et al. 2014a). In this study, the same PM_2.5_ exposure predictions were employed.

Briefly, we calibrated the AOD–PM_2.5_ relationship on each day of the study period (2003–2008) using data from grid cells with both ground PM_2.5_ monitors and AOD measurements (stage 1), and we used inverse probability weighting to address selection bias due to nonrandom missingness patterns in the AOD measurements. We then used the AOD–PM_2.5_ relationship to predict PM_2.5_ concentrations for grid cells that lacked monitors but had available AOD measurement data (stage 2). Finally, we used a generalized additive mixed model (GAMM) with spatial smoothing and a random intercept for each 1 km × 1 km grid cell to impute data for grid cells/days for which AOD measurements were not available (stage 3). The performance of the estimated PM_2.5_ was validated by 10-fold cross-validation. High out-of-sample *R*^2^ (*R*^2^ = 0.89, year-to-year variation 0.88–0.90 for the years 2003–2008) was found for days with available AOD data. Excellent performance held even in cells/days with no available AOD (*R*^2^ = 0.89, year-to-year variation 0.87–0.91 for the years 2003–2008). The 1-km model had better spatial (0.87) and temporal (0.87) out-of-sample *R*^2^ than the previous 10-km model (0.78 and 0.84, respectively). Details of the PM_2.5_ prediction models are in Kloog et al. (2014a).

Figure 1A shows an example of mean PM_2.5_ concentrations in 2004 at a 1 km × 1 km spatial resolution across New England. By averaging the estimated daily exposures at each location, we generated long-term exposures.

Figure 1B (a subset of the study area) shows that spatial variability existed even for daily data and was not identical to the long-term pattern shown in Figure 1A. That is, there was space–time variation in the PM_2.5_ exposure captured in this analysis, but not in previous time-series analyses.

Because the deaths were coded at the ZIP code level, both long- and short-term predictions were matched to ZIP codes by using ArcGIS (ESRI, Redlands, CA) and SAS (SAS Institute Inc., Cary, NC) to link the ZIP code centroid to the nearest PM_2.5_ grid.

Traditionally, studies of acute air pollution effects have controlled for temperature using values taken from the nearest airport. This approach is not feasible for the entire region because many residences are distant from airports. In addition, there is spatiotemporal variation in temperature. We have applied a similar 3-stage statistical modeling approach to estimate daily ambient temperature at 1 km × 1 km resolution in New England using satellite-derived surface temperature (Kloog et al. 2014b). To our knowledge, such fine control for temperature has not previously been used in air pollution epidemiology.

*Mortality data.* Individual mortality records were obtained from the U.S. Medicare program for all residents ≥ 65 years of age for all available years during 2003–2008 (CMS 2013b). The Medicare cohort was used because of the availability of ZIP code of residence data, whereas National Center for Health Statistics mortality data are only available at the county level. Additionally, previous studies found that elderly people are highly susceptible to the effects of particulate matter (Pope 2000). The Medicare beneficiary denominator file from the Centers for Medicare and Medicaid services (CMS 2013a) lists all beneficiaries enrolled in the Medicare fee-for-service (FFS) program and contains information on beneficiaries’ eligibility and enrollment in Medicare and the date of death. The Medicare Provider Analysis and Review (MEDPAR) file includes information on age, sex, race, ZIP code of residence, and one record for each hospital admission (CMS 2013c).

Daily mortality was first aggregated by ZIP code and then matched with the corresponding PM_2.5_ exposure. We summarized the mortality data by ZIP code and day because that was the finest resolution we could obtain for addresses. Because the mortality data sets did not include changes of residence, we assumed that the subjects lived at their current address over the entire study period.

*Covariates.* We used daily 1-km temperature data estimated from surface temperature measured by satellites (Kloog et al. 2014b). All socioeconomic variables were obtained through the U.S. Census Bureau 2000 Census Summary File 3, which includes social, economic, and housing characteristics (U.S. Census Bureau 2000). ZIP code tabulation area–level socioeconomic variables, including race, education, and median household income, were used. The county-level percentage of people who currently smoke every day, obtained from the CDC Behavioral Risk Factor Surveillance survey for the entire country, was also adjusted (CDC 2013). Dummy variables were used to control for day of the week.

*Statistical models.* Conventionally, the acute effects of air pollution are estimated by Poisson log-linear models, and the chronic effects of air pollution are estimated by Cox proportional hazard models (Kloog et al. 2013; Laden et al. 2006). Laird and Olivier (1981) noted the equivalence of the likelihood of a proportional hazard model with piecewise constant hazard for each year of follow-up and a Poisson regression with a dummy variable for each year of follow-up. We have taken advantage of this equivalence to generalize from dummy variables for each year to a spline of time to represent the baseline hazard and to aggregate subjects into counts per person time at risk, and we obtained a mixed Poisson regression model (Kloog et al. 2012). This approach allows the rate of death as a function of both long- and short-term exposures to be modeled simultaneously. By doing so, we achieved the equivalence of a separate time series analysis for each ZIP code, greatly reducing the exposure error in that part of the model, while simultaneously conducting a survival analysis on the participants, and we were also able to estimate the independent effects of both exposures.

Most time series studies have reported stronger associations with acute exposures when exposures were defined as the mean PM_2.5_ on the day of death and the previous day (lag01) than when they were defined as the mean PM_2.5_ on the current day only, or for exposures with longer lags (Schwartz et al. 1996; Schwartz 2004). We used the lag01 average for our main analysis but performed a sensitivity analysis on that choice. Long-term exposure was calculated as the 365-day moving average ending on the date of death so that our results were comparable with those of previous studies (Lepeule et al. 2012; Schwartz et al. 2008). Short-term exposure was defined as the difference between the 2-day average and the long-term average, ensuring that acute and chronic effects were independent. We subtracted the long-term average from the short-term average to avoid collinearity issues and to ensure that differences between ZIP codes in PM_2.5_ at a given time did not contribute to the short-term effect estimate. Thus, the short-term effect could not be confounded by variables that differed across ZIP codes.

Specifically, we fit a Poisson survival analysis with a logarithmic link function and a log (population) offset term and modeled the expected daily death counts (μ*_it_*) in the *i*th ZIP code on the *t*th day as follows:

log(*μ_it_*) = λ*_i_* + β_1_*PM_it_* + β_2_Δ*PM_it_* + λ(*t*) + temporal covariates + spatial covariates + offset, [1]

where λ*_i_* is a random intercept for each ZIP code, *PM_it_* is the 365-day moving average ending on day *t* in ZIP code *i*, Δ*PM_it_* is the deviation of the 2-day average from its long-term average (*PM_it_*) in ZIP code *i*, λ(*t*) is a smooth function of time, temporal covariates are temperature and day of the week, and spatial covariates are socioeconomic factors defined at the ZIP code level (percent of people without high school education, percent of white people, median household income) and smoking data at the county level. Additionally, a quasi-Poisson model was used to control for possible overdispersion (Ver Hoef and Boveng 2007).

We estimated λ(*t*) with a natural cubic spline with 5 degrees of freedom (df) per year to control for time and season trends. The specific temporal and spatial covariates that we used were a natural cubic spline for temperature with 3 df in total; a categorical variable for day of the week; linear variables for percent of people without high school education, percent of white people, median household income, and percent of people who currently smoke every day.

The number of deaths per ZIP code area over the study period (2003–2008) averaged 319 with a standard deviation of 430. Because the outcome was counts, we could not adjust for age and sex as in a Cox model. Instead, we adjusted for variables that varied by ZIP code. The analyses were repeated without mutual adjustment for short- and long-term PM_2.5_.

We modeled the association between all-cause mortality and PM_2.5_ at low doses in which the person-time at risk in each year of follow-up in each ZIP code was used as the offset. We also conducted effect modification by population size by choosing the median (4,628) of the ZIP code–level total population as the cutoff between urban and rural areas.

*Estimating the effects of low-level PM_2.5_.* For full cohort analyses with 10,938,852 person-years of follow-up, all observed deaths were used. To estimate effects at low levels of exposure, we performed restricted analyses: we conducted one analysis restricted to annual exposures < 10 μg/m^3^, below the current annual PM_2.5_ NAAQS of 12 μg/m^3^, and another restricted to observations with short-term exposure < 30 μg/m^3^, below the current daily PM_2.5_ NAAQS of 35 μg/m^3^. After these exclusions, the chronic analyses were restricted to 268,050 deaths out of 551,024 deaths in total, and the acute analyses were restricted to 422,637 deaths.

*Assessing the dose–response relationship.* For both the acute and chronic analyses, we fit penalized regression splines in the restricted analyses to estimate the shape of the dose–response curve below current U.S. EPA standards. The degrees of freedom of the penalized splines for PM_2.5_ were estimated by generalized cross-validation (GCV).

## Results

Table 1 presents a summary of the predicted exposures for both short- and long-term PM_2.5_ exposure across all grid cells in the study area.

**Table 1:** Descriptive statistics for PM_2.5_ exposure and temperature in New England, 2003–2008.

Covariate	Mean	SD	Minimum	Median	Maximum	Range	Q1	Q3	IQR
Lag01 PM_2.5 _(μg/m^3^)	8.21	5.10	0.00	7.10	53.98	53.98	4.60	10.65	6.05
1-year PM_2.5 _(μg/m^3^)	8.12	2.28	0.08	8.15	20.22	20.14	6.22	10.00	3.78
Temperature (˚C)	9.24	6.50	–36.79	9.81	41.51	78.30	4.90	14.39	9.49

Table 2 presents the estimated percent change in all-cause mortality with 95% CIs for a 10-μg/m^3^ increase in both short- and long-term PM_2.5_ in the restricted and full cohort. In the restricted population, we found an estimated 9.28% increase in mortality (95% CI: 0.76, 18.52%) for every 10-μg/m^3^ increase in long-term PM_2.5_ exposure. A 2.14% increase in mortality (95% CI: 1.34, 2.95%) was observed for every 10-μg/m^3^ increase in short-term PM_2.5_ exposure. For long-term exposure, the effect estimates were smaller when higher pollution days were included (7.52%; 95% CI: 1.95, 13.40%), suggesting larger effects between low-concentration long-term PM_2.5_ and mortality.

**Table 2:** Percent increase in mortality (95% CI) for a 10-μg/m^3^ increase for both short-term and long-term PM_2.5_.

PM_2.5_ exposure	Model	Percent increase	*p*-Value
With mutual adjustment
Short-term PM_2.5_	Low daily exposure^*a*^	2.14 ± 0.81	< 0.001
	Full cohort	2.14 ± 0.75	< 0.001
Long-term PM_2.5_	Low chronic exposure^*b*^	9.28 ± 8.88	0.032
	Full cohort	7.52 ± 5.73	0.007
Without mutual adjustment
Short-term PM_2.5_	Low daily exposure^*a*^	2.07 ± 0.80	< 0.001
	Full cohort	2.08 ± 0.76	< 0.001
Long-term PM_2.5_	Low chronic exposure^*b*^	7.16 ± 8.75	0.109
	Full cohort	6.46 ± 5.69	0.026
The full cohort analysis had 551,024 deaths. ^***a***^The analysis was restricted only to person time with daily PM_2.5_ < 30 μg/m^3^ (422,637 deaths). ^***b***^The analysis was restricted only to person time with chronic PM_2.5_ < 10 μg/m^3^ (268,050 deaths).			

Without mutual adjustment, lower estimates were found for both acute and chronic effects than for those with mutual adjustment. In full-cohort analyses, a 2.08% (95% CI: 1.32, 2.84%) and a 6.46% (95% CI: 0.93, 12.30%) increase in mortality was found for each 10-μg/m^3^ increase in short- and long-term PM_2.5_, respectively. In restricted analyses, the corresponding effect estimates were 2.07% (95% CI: 1.27, 2.89%) and 7.16% (95% CI: –1.23, 16.27%), respectively.

**Figure 2:**
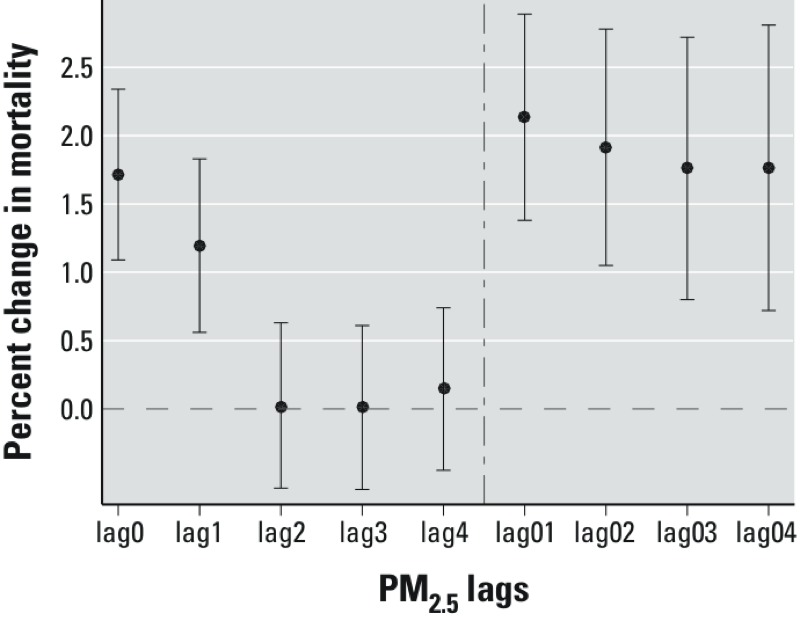
Percent change in mortality per 10-μg/m^3^ increase in short-term PM_2.5_ with different lags with mutual adjustment. Error bars indicate the 95% CIs.

Our results were robust to the choice of lag period for acute exposure. We analyzed different averaging periods (Figure 2): for example, lag0 (day of death exposure) and lag04 (a moving average of day of death exposure and previous 4-day exposure). For the acute effects, we found a significant but smaller association for lag0 PM_2.5_ (1.71%; 95% CI: 1.09, 2.34%) and lag04 PM_2.5_ (1.76%; 95% CI: 0.72, 2.81%) than for lag01 analysis. The lag period used for short-term exposure did not affect estimates of chronic effects. For example, estimated increases in mortality with a 10-μg/m^3^ increase in long-term PM_2.5_ were 7.35% (95% CI: 1.79, 13.21%) and 7.25% (95% CI: 1.69, 13.12%) when short-term PM_2.5_ was classified using lag0 or lag04, respectively.

We also examined effect modification by population size. In the full cohort, a significant interaction was found for chronic effects (*p* < 0.01), with a larger effect of 12.56% (95% CI: 5.71, 19.85%) in urban areas compared with 3.21% (95% CI: –2.92, 9.72%) in rural areas. Such a significant interaction, however, was not observed in the restricted analysis (*p* = 0.16). Estimates were 14.27% (95% CI: 3.19, 26.53%) and 5.48% (95% CI: –4.21, 16.16%) in urban and rural areas, respectively. For short-term exposure, population size did not modify the acute effects in either the full cohort or the restricted analysis (*p* = 0.74 and 0.46, respectively).

**Figure 3:**
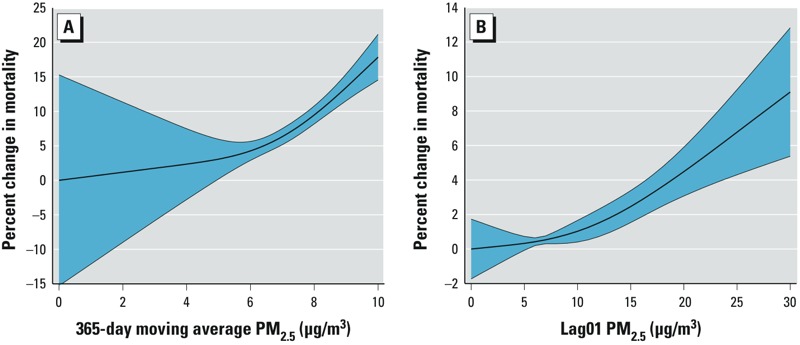
The dose–response relationship between long-term PM_2.5_ and mortality at low doses with mutual adjustment (*A*) and the dose–response relationship between short-term PM_2.5_ and mortality at low doses without mutual adjustment (*B*). Shaded areas indicate the 95% CIs.

In our penalized spline model for long-term exposure below the cutoff of 10 μg/m^3^ (Figure 3A), we found a nonlinear relationship between long-term PM_2.5_ and mortality. The association was linear with evidence of a smaller effect < 6 μg/m^3^. However, a large confidence interval was observed; hence, we could not be confident whether the slope of the dose–response curve changed for long-term exposures < 6 μg/m^3^. When examining the shape of the dose–response curve for chronic effects, both a linear term for short-term exposure (the difference) and a penalized spline for long-term average exposure were included in the model, resulting in a penalized spline with a df of 1.71. In contrast, we only included the 2-day average in the penalized spline model of acute effects in order to provide an interpretable dose–response relationship (Figure 3B). The results of this analysis indicated a linear association across the exposure distribution, but we could not be certain about the shape of the slope for acute effects < 3 μg/m^3^.

## Discussion

When we applied the predicted daily PM_2.5_ with 1-km spatial resolution from our novel hybrid models, we observed that both short- and long-term PM_2.5_ exposure were significantly associated with all-cause mortality among residents of New England ≥ 65 years of age, even when restricted to ZIP codes and times with annual exposures < 10 μg/m^3^ or with daily exposure < 30 μg/m^3^. Hence, the association of particle exposure with mortality exists for concentrations below the current standards established by the United States, the World Health Organization (WHO) (10 μg/m^3^ of annual average PM_2.5_, 25 μg/m^3^ daily), and the European Union (EU) (25 μg/m^3^ of annual average PM_2.5_) (EU 2013; WHO 2013). Notably, this analysis includes all areas in New England and all Medicare enrollees ≥ 65 years of age in this region, and it provides chronic effect estimates that are independent of acute effects. Based on a penalized spline model, the positive dose–response relationship between chronic exposure and mortality appears to be linear for PM_2.5_ concentrations ≥ 6 μg/m^3^, with a positive (though smaller and less precise) dose–response slope continuing below this level. This lack of power is likely due to the small exposed population in areas with annual PM_2.5_ < 6 μg/m^3^, which were quite rural.

For acute effects, we found a 2.14% (95% CI: 1.38, 2.89%) increase in all-cause mortality per 10-μg/m^3^ increment in PM_2.5_ for the full cohort of our study, which is higher than the effect size of most studies using city averages obtained from monitors. For instance, in a U.S. national study by Zanobetti and Schwartz (2009), the effect size was 0.98% (95% CI: 0.75, 1.22%). Similar results were also obtained in a systematic review, where researchers determined that the overall summary estimate was 1.04% (95% CI: 0.52, 1.56%) per 10-μg/m^3^ increment in PM_2.5_ (Atkinson et al. 2014). The exposure data used in most previous studies had low spatial resolution (citywide average, not ZIP code), which introduced exposure measurement error and likely resulted in a downward bias in estimates; our results (for the acute effect) are consistent with such a phenomenon. Our restricted study estimated a 2.14% (95% CI: 1.34, 2.95%) increase in all-cause mortality per 10-μg/m^3^ increment in PM_2.5_, which was close to the effect size of the full cohort study, possibly because the sample size of the restricted study for acute effects was close to that of the full cohort. Furthermore, the U.S. EPA daily standard (35 μg/m^3^) was almost never exceeded in this study. In addition, lower effect estimates for short-term exposure were observed with mutual adjustment for both full cohort and restricted analyses. This finding has important implications for the interpretation of previous studies without such mutual adjustment.

For chronic effects, the effect estimate in our full cohort study was consistent with findings of previous studies with comparable sample sizes (Hoek et al. 2013; Laden et al. 2006; Lepeule et al. 2012). For example, an ACS study comprising 500,000 adults from 51 U.S. cities reported a 6% (95% CI: 2, 11%) increase in all-cause mortality for each 10-μg/m^3^ increment in PM_2.5_ (Pope et al. 2002). A study of 13.2 million elderly Medicare recipients across the eastern United States found a 6.8% (95% CI: 4.9, 8.7%) increase in all-cause mortality for each 10-μg/m^3^ increment in PM_2.5_ (Zeger et al. 2008). When we restricted our analysis to annual concentrations < 10 μg/m^3^, a larger slope of 9.28% (95% CI: 0.76, 18.52%) increase per 10 μg/m^3^ was observed. Our findings suggest a larger effect at low concentrations among those ≥ 65 years of age, which may also reflect particle composition. The sources and composition of the particles may differ between low-pollution days and high-pollution days, which are likely more affected by secondary aerosols. Compared with the effect estimate for the full cohort, the effect estimate from the restricted analysis was closer to estimates published in the literature that reported larger effect estimates, such as those reported by the ESCAPE (European Study of Cohorts for Air Pollution Effects) study, the Harvard Six Cities study, and the Women’s Health Initiative study (Beelen et al. 2014; Puett et al. 2008). Smaller effect estimates were also observed for chronic effects without mutual adjustment.

To the best of our knowledge, this study is the first of its kind to restrict exposure and to explore the dose–response relationship between PM_2.5_ below the current U.S. EPA standards (12 μg/m^3^ of annual average PM_2.5_, 35 μg/m^3^ daily) and mortality. Moreover, the use of the Medicare cohort means that we studied the entire population of Medicare enrollees ≥ 65 years of age and not a convenience sample. In addition, temperature was controlled on a 1 km × 1 km fine geographic scale. The acute and chronic effects observed in analyses restricted to low PM_2.5_ exposure were similar to or even higher than those of the full cohort analyses. These results indicate that the adverse health effects of PM_2.5_ are at least retained, if not strengthened, at low levels of exposure. However, the findings from the penalized spline model did not support a strong association at the lowest range of PM_2.5_ concentrations. This finding provides epidemiological evidence for the reevaluation of U.S. EPA guidelines and standards, although more evidence is needed to confirm the association < 6 μg/m^3^.

The Poisson survival analysis applied in this study provided a novel method of simultaneously assessing acute and chronic effects. As shown in our analysis, the chronic effect estimate was much larger than the acute effect estimate after controlling for the acute estimate, indicating that there were chronic effects of PM_2.5_, which cannot be solely explained by the short-term exposure.

Another key component of this study is that the application of high spatial (1 km × 1 km) and temporal (daily) resolution of PM_2.5_ concentrations reduced exposure error to a certain extent. The out-of-sample *R*^2^ was higher than that for the predictions with 10 km × 10 km spatial resolution.

A potential limitation is the limited availability of individual-level confounders, such as smoking status, which could bias the health effect estimates. We were able to control for ZIP code–level education, median income, race, and county-level smoking data. However, Brochu et al. (2011) reported that census tract–level socioeconomic indicators were uncorrelated with PM_2.5_ on the subregional and local scale, providing some assurance that confounding by socioeconomic status may not be much of an issue. The results reported by Brochu et al. (2011) suggest that those variables may not confound the association, but the inability to control for them remains an issue. Another limitation is that we did not examine other pollutants such as ozone (O_3_) or nitrogen dioxide (NO_2_) owing to a lack of data at the same spatial level as that of PM_2.5_.

## Conclusions

In conclusion, the acute and chronic effects of low-concentration PM_2.5_ were examined for a Medicare population using a comprehensive exposure data set obtained from a satellite-based prediction model. Our findings show that both short- and long-term exposure to PM_2.5_ were associated with all-cause mortality, even for exposure levels not exceeding the newly revised U.S. EPA standards, suggesting that adverse health effects occur at low levels of fine particles. The policy implication of these findings is that improving the air quality at even lower levels of PM_2.5_ than presently allowed by the U.S. EPA standards can yield health benefits.
